# Blood-based biomarkers: diagnostic value in brain tumors (focus on gliomas)

**DOI:** 10.3389/fneur.2023.1297835

**Published:** 2023-10-23

**Authors:** Yuting Yang, Fei Hu, Song Wu, Zhangliang Huang, Kun Wei, Yuan Ma, Qing Ou-Yang

**Affiliations:** ^1^Institute of Biomedical Engineering, College of Medicine, Southwest Jiaotong University, Chengdu, Sichuan, China; ^2^Department of Neurosurgery, Affiliated Hospital of Southwest Jiaotong University, The General Hospital of Western Theater Command, Chengdu, Sichuan, China

**Keywords:** brain tumors, glioma, pan-immune-inflammation value, neutrophil–lymphocyte ratio, diagnostic indicator

## Abstract

**Background:**

Brain tumors, especially gliomas, are known for high lethality. It is currently understood that the correlations of tumors with coagulation and inflammation have been gradually revealed.

**Objective:**

This study aimed to explore the potential value of several reported peripheral blood parameters as comprehensively as possible, with preoperative diagnosis and identification of brain tumors (focus on gliomas).

**Methods:**

Patients with central nervous system tumors (craniopharyngioma, ependymoma, spinal meningioma, acoustic neuroma, brain metastases, meningioma, and glioma) or primary trigeminal neuralgia admitted to our hospital were retrospectively analyzed. The results of the routine coagulation factor test, serum albumin test, and blood cell test in peripheral blood were recorded for each group of patients on admission. Neutrophil–lymphocyte ratio (NLR), derived NLR (dNLR), platelet–lymphocyte ratio (PLR), lymphocyte–monocyte ratio (LMR), prognostic nutritional index (PNI), the systemic immune-inflammation index (SII), pan-immune-inflammation value (PIV), and their pairings were calculated. Their ability to identify brain tumors and their correlation with glioma grade were analyzed.

**Results:**

A total of 698 patients were included in this retrospective case–control study. Glioma patients had higher NLR, SII, and PIV but lower LMR. The NLR in the brain metastasis group was lower than that in the control, meningioma, and acoustic neuroma groups, but the SII and PIV were higher than those in the ependymoma group. Fibrinogen, white blood cell count, neutrophil count, NLR, SII, and PIV in the GBM group were higher than those in the control group. In all comparisons, NLR and NLR + dNLR showed the greatest accuracy, with areas under the curve (AUCs) of 0.7490 (0.6482–0.8498) and 0.7481 (0.6457–0.8505), respectively. PIV, dNLR + PIV, and LMR + PIV ranked second, with AUCs of 0.7200 (0.6551–0.7849), 0.7200 (0.6526–0.7874), 0.7204 (0.6530–0.7878) and 0.7206 (0.6536–0.7875), respectively.

**Conclusion:**

NLR, PIV, and their combinations show high sensitivity and specificity in the diagnosis of brain tumors, especially gliomas. Overall, our results provide evidence for these convenient and reliable peripheral blood markers.

## Introduction

Approximately 300, 000 people worldwide are diagnosed with brain tumors each year, and approximately 250, 000 died (1). According to the classification published by the WHO, common central nervous system (CNS) tumors include acoustic neuroma, meningioma, brain metastases, glioma, and some other tumors (2). The most common primary brain tumor is meningioma (39% of all brain tumors and 54.5% of non-malignant brain tumors), followed by tumors of the saddle area (craniopharyngioma, pituitary tumors, etc.) (3). Among primary malignant tumors of the brain, glioblastoma (GBM) has the highest incidence [14.3% of all tumors, 49.1% of malignant tumors, and 81% of glioma (4)], with a five-year survival rate of only 6.8% (3). The incidence of ependymoma is approximately 0.2 to 0.4 per 100, 000 individuals (5). The treatment of brain tumors includes surgery, radiotherapy, and chemotherapy (temozolomide adjuvant chemotherapy). Most patients died of progressive disease. Thus, accurate grading has a huge impact on the way of treatment.

The identification of brain tumors has long been based on histological examination (the patient undergoes surgery, and the diagnosis is confirmed by a pathologist). Sometimes clinical presentation and radiological methods (visualization with contrast-enhanced magnetic resonance imaging, X-rays, etc.) can also make a simple distinction. However, histological and radiological tests are invasive and expensive. Recently, liquid biopsies based on circulating tumor cells (CTCS) in peripheral blood samples have been recognized as superior technological advances (6), but the test remains expensive, and routine screening is not available in most institutions. We still lack more economical, convenient, and widely available diagnostic biomarkers.

Cancer has long been reported to be associated with chronic inflammation (7). Recently, some peripheral blood-based indicators, neutrophil–lymphocyte ratio (NLR), derived NLR (dNLR), platelet–lymphocyte ratio (PLR), lymphocyte–monocyte ratio (LMR), prognostic nutritional index (PNI), the systemic immune–inflammation index (SII), and pan–immune–inflammation value (PIV) have been reported to be associated with the prognosis or stratification of several tumors, such as glioma (8–10), lung cancer (11), and colorectal cancer (12–14). However, only a few studies have reported their diagnostic value in brain tumors, particularly glioma (15, 16). At the same time, tumor patients are characterized by a dysregulated coagulation system and a systemic hypercoagulable state (17). Different degrees of activation of the coagulation system seem to be associated with tumor aggressiveness (18). Therefore, some coagulation parameters and inflammatory indicators may be valuable in tumor diagnosis. Among them, PIV, a novel immune indicator recently created, has been shown to have an independent and significant association with poor outcomes in GBM patients, who received postoperative radiotherapy and concomitant addition of temozolomide adjuvant therapy (19).

In this study, we compared differences in coagulation parameters, serum albumin levels, and peripheral blood cell counts among primary trigeminal neuralgia, ependymoma, craniopharyngioma, acoustic neuroma, brain metastases, meningiomas, and gliomas. Furthermore, the diagnostic value of NLR, dNLR, PLR, LMR, PNI, SII, PIV, and their combinations in brain tumors was further evaluated, especially in GBM.

## Methods

### Study design

A descriptive case–control design was adopted, and to ensure the research quality, the STROBE checklist was used to report findings (Supplementary Table S1).

### Setting

The medical records of patients with brain tumors (craniopharyngioma, ependymoma, spinal meningioma, acoustic neuroma, brain metastases, meningioma, or glioma) and trigeminal neuralgia (NT) patients admitted to the Neurosurgery Department of the General Hospital of Western Theater Command in Chengdu from January 2017 to December 2022 were retrospectively analyzed.

### Participants

Patients included in this study had to meet the following criteria: (1) craniopharyngioma, ependymoma, spinal meningioma, acoustic neuroma, brain metastases, and meningioma, or glioma confirmed by biopsy or postoperative pathological examination; (2) complete preoperative routine coagulation parameters, serum albumin level, and peripheral blood cell count data; (3) no previous hypertension, hyperlipidemia, diabetes, metabolic syndrome, heart disease, liver and kidney dysfunction, hematologic disorders, autoimmune diseases, no preoperative fever, infectious diseases, and no use of preoperative anti-infective drugs and steroids; (4) no previous brain tumors, currently has only one type of brain tumor and no tumor-specific treatment history such as radiotherapy or chemotherapy (except brain metastases); (5) informed consent.

As for the control group, patients admitted to our neurosurgery department for trigeminal nerve microvascular decompression or facial nerve microvascular decompression during the same period, and the requirements were as follows: (1) complete preoperative information on routine coagulation parameters, serum albumin levels, and peripheral blood cell counts; (2) no previous tumor, hypertension, hyperlipidemia, diabetes, metabolic syndrome, heart disease, liver and kidney dysfunction, hematologic disorders, autoimmune diseases, no preoperative fever, infectious diseases, and no anti–infective drugs; (3) informed consent.

### Data collection

Demographic parameters and pathological information were retrieved and recorded from the medical record, including gender, age, diagnosis, tumor grade, histological type, and primary site of brain metastasis. Patients were routinely examined upon admission for coagulation parameters [prothrombin time (PT), fibrinogen (FIB) level, activated partial thromboplastin time (APTT), and thrombin time (TT)], serum albumin levels, and peripheral blood cell counts [platelet count, white blood cell (WBC) count, neutrophil count, lymphocyte count, monocyte count, eosinophil count, and basophil count]. All tests were performed at our hospital testing department.

### Data measurement

In addition, the above data were used to calculate NLR (neutrophil/lymphocyte count), dNLR ([white blood cell count – neutrophil count]/lymphocyte count), PLR (platelet count/lymphocyte count), LMR (lymphocyte count/monocyte count), PNI (albumin count + lymphocyte count ^*^5), SII (platelet count ^*^ neutrophil count/lymphocyte count), and PIV (neutrophil count ^*^ platelet count ^*^ monocyte count/lymphocyte count). Furthermore, these data were used to calculate NLR + dNLR, NLR + PLR, NLR + LMR, NLR + PNI, NLR + SII, NLR + PIV, dNLR + PLR, dNLR + LMR, dNLR + PNI, dNLR + SII, dNLR + PIV, PLR + LMR, PLR + PNI, PLR + SII, PLR + PIV, LMR + PNI, LMR + SII, LMR + PIV, PNI + SII, PNI + PIV, and SII + PIV.

### Statistical analysis

Statistical analysis was performed using GraphPad Prism 9.4.1. First, the Kruskal–Wallis test was used to analyze the normality of the variables. We used the median (range) to represent all data. The Mann–Whitney *U*-test was used for comparison between groups. The Spearman correlation test was used to analyze the correlation among variables. The diagnostic efficacy of peripheral blood inflammatory markers in subjects was evaluated by the area under the curve (AUC) obtained from the receiver operating characteristic (ROC) curve. A *P*-value of <0.05 was considered to be statistically significant.

## Results

### Participants' characteristics

**Figure 1:**
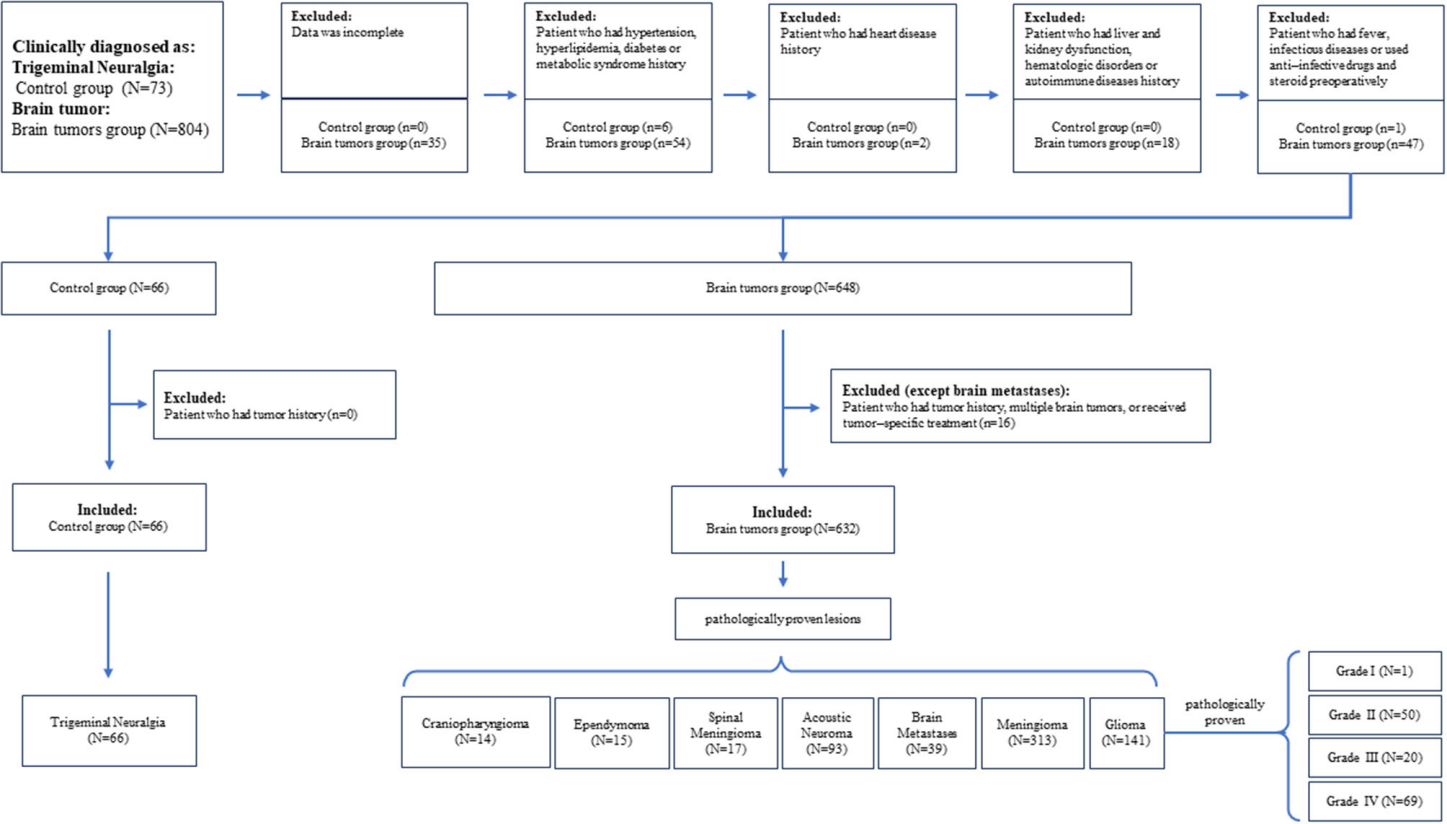
Selection flowchart of participants.

A total of 698 patients were included in this study, including 66 patients with trigeminal neuralgia, 14 patients with craniopharyngioma, 15 patients with ventricular meningioma, 17 patients with chordoma, 93 patients with auditory neuroma, 39 patients with brain metastases, 313 patients of meningioma, and 141 patients with glioma (grade I, 1 case; grade II, 50 cases; grade III, 20 cases; and grade IV, 69 cases). The selection flowchart is demonstrated in Figure 1.

Glioma patients [48 (8–74)] were significantly younger than control patients [58.5 (19–82)], acoustic neuroma patients [54.5 (15–83)], brain metastases patients [59 (39–78)], and meningioma patients [53 (5–81)]. Patients in the meningioma group were also significantly younger than the control group. The majority of patients with meningioma were female [232, (74.12%)]. Detailed demographic information is listed in Table 1.

**Table 1:** Preoperative characteristics of patients with brain tumors.

**Parameter**	**Trigeminal neuralgia**	**Craniopharyngioma**	**Ependymoma**	**Spinal meningioma**	**Acoustic neuroma**	**Brain metastases**	**Meningioma**	**Glioma**
Age	58.5 (19–82)	49 (19–66)	47.5 (4–76)	55.5 (22–83)	54.5 (15–83)	59 (39–78)	53 (5–81)^*^	48 (8–74)^*+§#^
No. of patients	66	14	15	17	93	39	313	141
Male (*n*, %)	27 (40.91%)	6 (42.86%)	6 (40%)	14 (82.35%)	36 (38.71%)^‡^	19 (48.72%)	81 (25.88%)^‡^	85 (60.28%)^+§^
Female (*n*, %)	39 (59.09%)	8 (57.14%)	9 (60%)	3 (17.65%)	57 (61.29%)^‡^	20 (51.28%)	232 (74.12%)^‡^	56 (39.72%)^+§^
Albumin (g/L)	42.9 (37.3–49.6)	42.95 (39.6–46.4)	44.4 (31.2–49.9)	42.8 (39.9–53.9)	43.3 (34–54)	41.9 (31.8–50)	42.8 (34.9–53.2)	43 (35.1–55)
PT (s)	10.6 (9–12.9)	10.25 (9.8–12)	10.7 (9.6–12.5)	10.5 (9.6–11.5)	10.6 (9.2–12.3)	10.8 (9.4–12.4)	10.6 (8.7–13.3)	10.7 (9–13.2)
FIB (g/L)	2.35 (1.71–6.5)	2.58 (1.87–5.34)	2.58 (2.11–5.1)	2.63 (1.86–3.77)	2.59 (1.52–4.86)	2.63 (2–5.6)	2.63 (1.18–9.27)	2.56 (1.62–6.85)
APTT (s)	26.2 (18.1–42.6)	25.35 (21.6–34.7)	29.3 (22.8–46.5)	28 (23.2–42)	26.8 (19.2–32.3)	25.8 (19.6–33.6)	26.3 (15.1–39.3)	25.95 (17.4–36)
TT (s)	17.8 (15.3–22.3)	18.9 (16.2–21.9)	17.9 (11.72–20.2)	18.2 (16.6–20.3)	17.8 (15.3–25.9)	18.3 (16.1–20.6)	18 (15.1–21.6)	18.15 (14.1–23.6)
Platelets (10^∧^9/L)	166 (88–314)	155 (113–243)	204 (110–402)	190 (118–254)	180 (74–406)	179.5 (50–395)	174 (64–517)	188 (83–426)
WBCs (10^∧^9/L)	5.41 (3.3–14.62)	6.32 (3.53–11.33)	7.11 (4.57–11.33)	5.53 (3.82–10.78)	5.56 (3.22–16.7)	6.24 (2.61–21.48)	5.65 (3.08–20.88)	6.82 (3–15.26)^*+#^
Neutrophils (10^∧^9/L)	3.48 (1.57–13.79)	3.72 (1.75–10.05)	4.03 (2.17–12.76)	3.46 (2.12–7.41)	3.46 (1.69–7.93)	4.43 (1.71–13.98)^*+^	3.57 (1.32–12.5)^§^	4.35 (1.8–14.37)^*+#^
Lymphocytes (10^∧^9/L)	1.48 (0.74–3.01)	2.04 (0.63–2.75)	1.68 (1–5.54)	1.57 (0.94–2.81)	1.62 (0.76–2.9)	1.28 (0.42–2.69)^∧^	1.55 (0.37–4.34)	1.56 (0.49–3.19)
Monocytes (10^∧^9/L)	0.34 (0.06–0.77)	0.32 (0.2–0.6)	0.34 (0.24–0.79)	0.36 (0.16–0.79)	0.32 (0.12–0.75)	0.35 (0.09–1.2)	0.34 (0.13–1.2)	0.39 (0.03–1.31) ^+#^
Eosinophils (10^∧^9/L)	0.12 (0–0.66)	0.18 (0.03–0.45)	0.12 (0–0.46)	0.1 (0.02–0.23)	0.11 (0–0.62)	0.07 (0–0.67)^∧^	0.1 (0–0.8)	0.08 (0–0.92)^∧#^
Basophils (10^∧^9/L)	0.02 (0–0.08)	0.02 (0–0.1)	0.02 (0.01–0.07)	0.03 (0.01–0.07)	0.02 (0–0.08)	0.02 (0.01–0.07)	0.02 (0–0.13)	0.02 (0–0.08)
NLR	2.11 (0.97–18.39)	1.74 (0.9–15.95)	2.22 (0.93–8.92)	2.13 (0.84–4.29)	2.11 (0.83–4.79)	3.65 (1.2–16.38)^*∧+^	2.13 (0.6–18.89)^§^	2.81 (0.97–28.74)^*∧+#^
dNLR	1.32 (1.11–2.32)	1.28 (1.16–2.03)	1.29 (1.11–1.58)	1.27 (1.21–1.75)	1.3 (1.14–1.72)	1.34 (1.09–1.97)	1.29 (1.1–2.32)	1.33 (1.1–2.13)
PLR	107.8 (48.81–409.5)	75.58 (43.64–350.8)	87.48 (40.2–226)	134(63.6–204.8)	110.09 (44.71–476.32)	127.8 (27.59–278.6)	110.32 (33.33–395.24)	119.48 (41.25–776)
LMR	4.42 (0.96–12.5)	6.4 (1.05–8.59)	4.75 (2.64–10.45)	5.64 (2.24–7.81)	4.71 (1.72–9.83)	3.87 (0.38–12.56)^∧+^	4.91 (0.92–12.06)^§^	4.02 (0.92–16.33)^∧+#^
PNI	50.2 (41–60.6)	54.17 (44.55–56.85)	52.45 (39.65–75.5)	52.6 (45.3–60.1)	51.7 (40.55–62.35)	49.7 (33.9–58.75)	51.3 (41.4–66.15)	51.1 (40.55–66.65)
SII	366.67 (151.31–3934.75)	245.08 (106.6–3525.48)	407 (130.25–1684.7)	420.2 (145.65–1089.74)	369.77 (144.46–1733.79)	545.5 (18.97–3411.13)^∧^	358.5 (86.23–3235.63)	514.8 (152.58–3142.07)^∧+#^
PIV	124.8 (26.99–2462)	92.82 (35.18–2115)	157.7 (60.66–551.5)	114.1 (57.91–590.4)	131.0 (26.37–603.4)	222.2 (23.54–3445)^∧^	120.8 (21.72–2394)^§^	196.5 (21.63–4126)^*∧+#^

Values are median (range). ^*^p < 0.05, compared to trigeminal neuralgia. ^∧^p < 0.05, compared to craniopharyngioma. ^†^p < 0.05, compared to ependymoma. ^‡^p < 0.05, compared to spinal meningioma. ^+^ p < 0.05, compared to acoustic neuroma. ^§^p < 0.05, compared to brain metastases. ^#^p < 0.05, compared to meningioma.

### Comparison of preoperative blood markers between the control group and the tumor group

**Figure 2:**
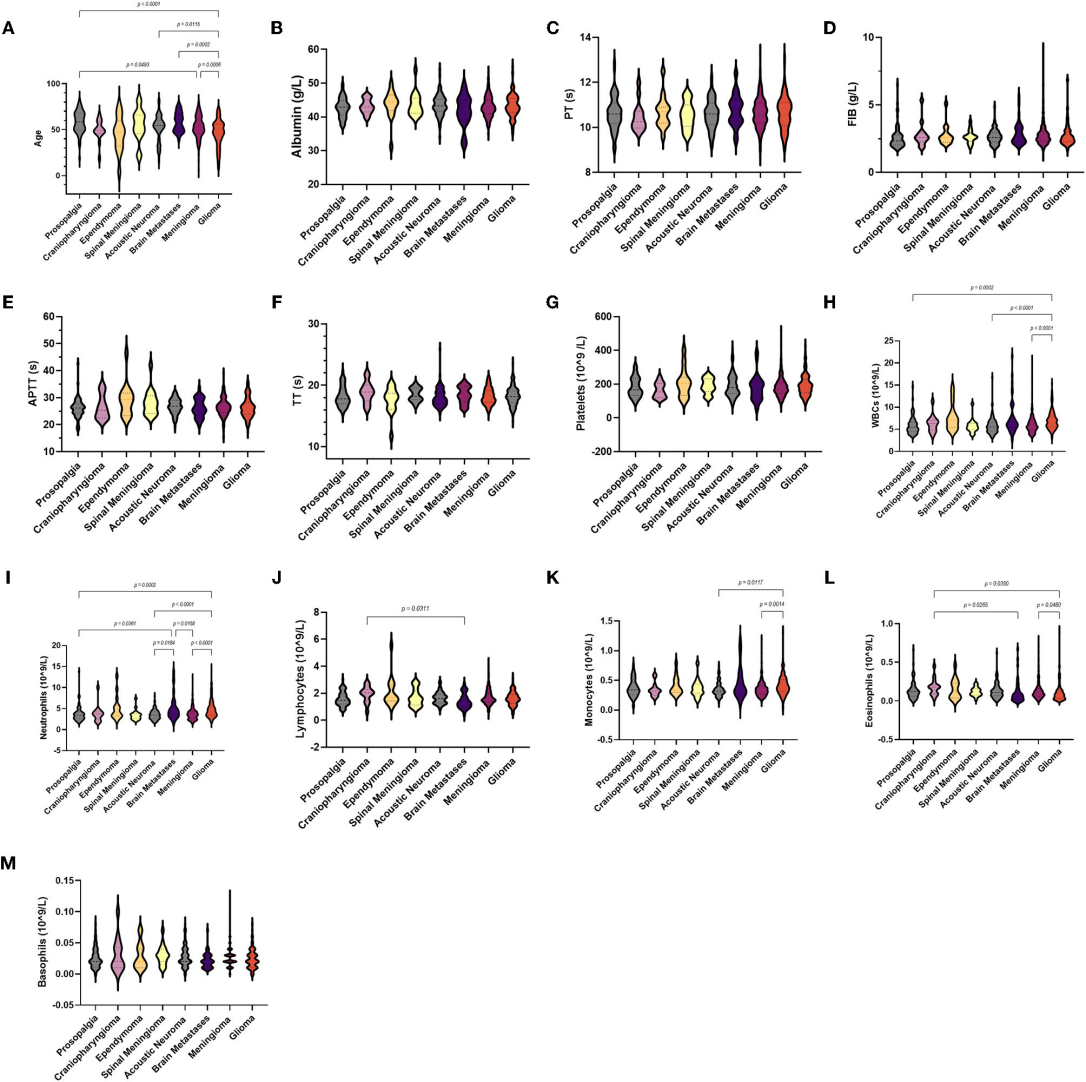
Violin diagram showing comparative results of characteristics in the trigeminal neuralgia group, craniopharyngioma group, ependymoma group, acoustic neuroma group, brain metastases group, meningioma group, and glioma group (the dashed line in the middle represents the median and the dashed lines on both sides represent the interquartile range). **(A)** Age, **(B)** albumin, **(C)** PT, **(D)** FIB, **(E)** APTT, **(F)** TT, **(G)** platelets, **(H)** WBCs, **(I)** neutrophils, **(J)** lymphocytes, **(K)** monocytes, **(L)** eosinophils, **(M)** basophils.

In all groups, no significant differences were observed in albumin, basophil count, and coagulation parameters (Figure 2). For neutrophils, the brain metastasis group [4.43 (1.71–13.98)] was higher than the trigeminal neuralgia [3.48 (1.57–13.79)], acoustic neuroma [3.46 (1.69–7.93)], and meningioma groups [4.43 (1.71–13.98)]. Compared with the control group, the acoustic neuroma group, the meningioma group, and the glioma group had much higher white blood cell counts [6.82 (3–15.26)] and neutrophil counts [4.35 (1.8–14.37)]. Meanwhile, the monocyte count of glioma patients [0.39 (0.03–1.31)] was higher than that of acoustic neuroma [0.32 (0.12–0.75)] and meningioma patients [0.34 (0.13–1.2)].

**Figure 3:**
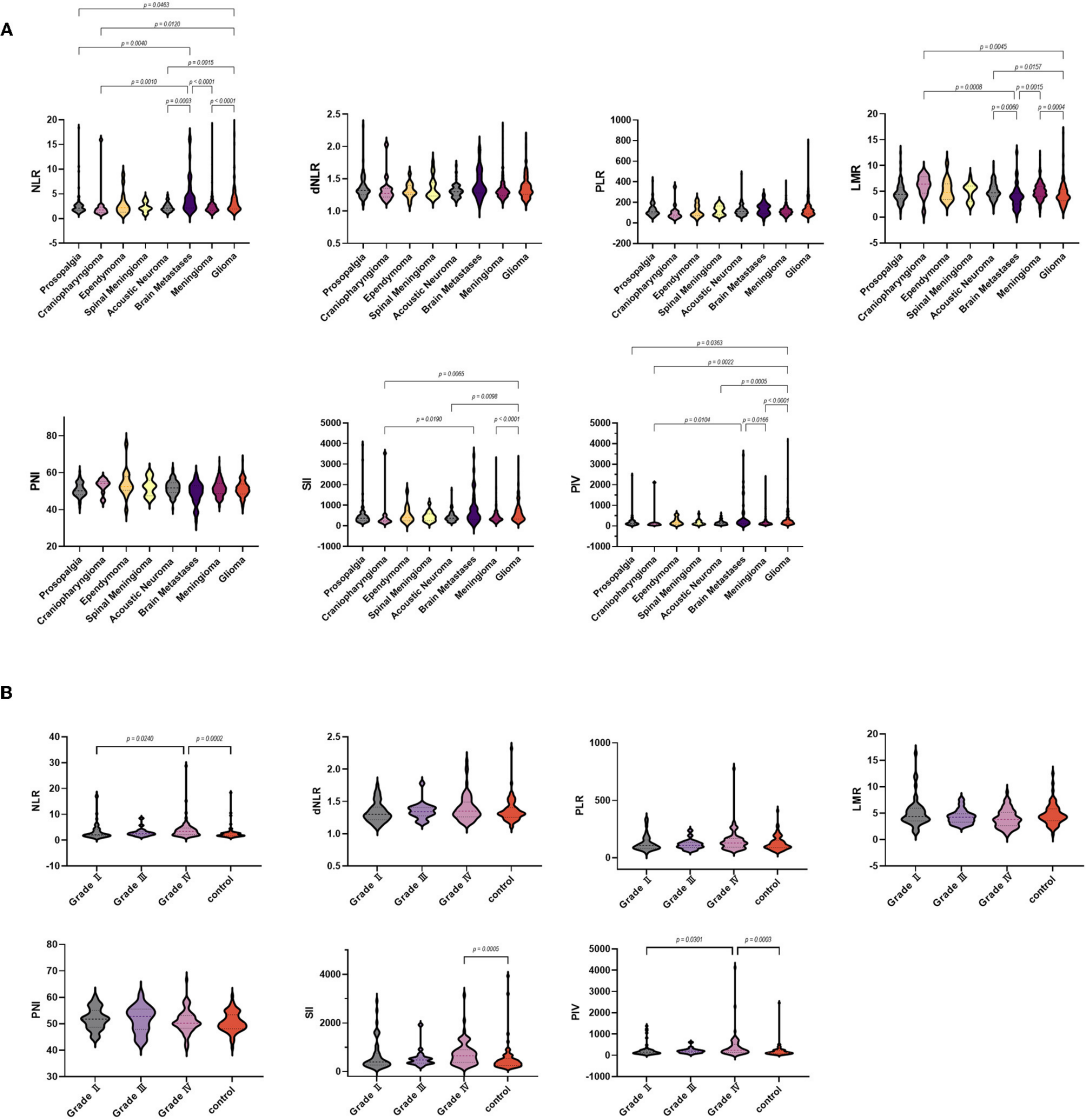
Violin diagram showing comparative results of preoperative inflammatory markers in different groups (the dashed line in the middle represents the median and the dashed lines on both sides represent the interquartile range). **(A)** Comparison of preoperative blood markers between the control group and the tumor group, **(B)** Comparison of preoperative blood markers for glioma of different grades.

As for laboratory parameters (Figure 3A), NLR and PIV were higher in the brain metastasis group than in the acoustic neuroma group, but the data were not significant. The NLR, SII, and PIV of glioma patients [2.81 (0.97–28.74), 514.8 (152.58–3142.07), and 196.5 (21.63–4126)] were significantly higher than the trigeminal neuralgia, craniopharyngioma, acoustic neuroma, and meningioma. We also observed lower LMR in the glioma group [4.02 (0.92–16.33)]. Moreover, NLR was lower in the brain metastasis group than in the control, meningioma, and acoustic neuroma groups, but the SII and PIV were higher than in the ependymoma group. Surprisingly, there were no differences in dNLR, PLR, and PNI among all groups.

### Comparison of preoperative blood markers for glioma of different grades

**Figure 4:**
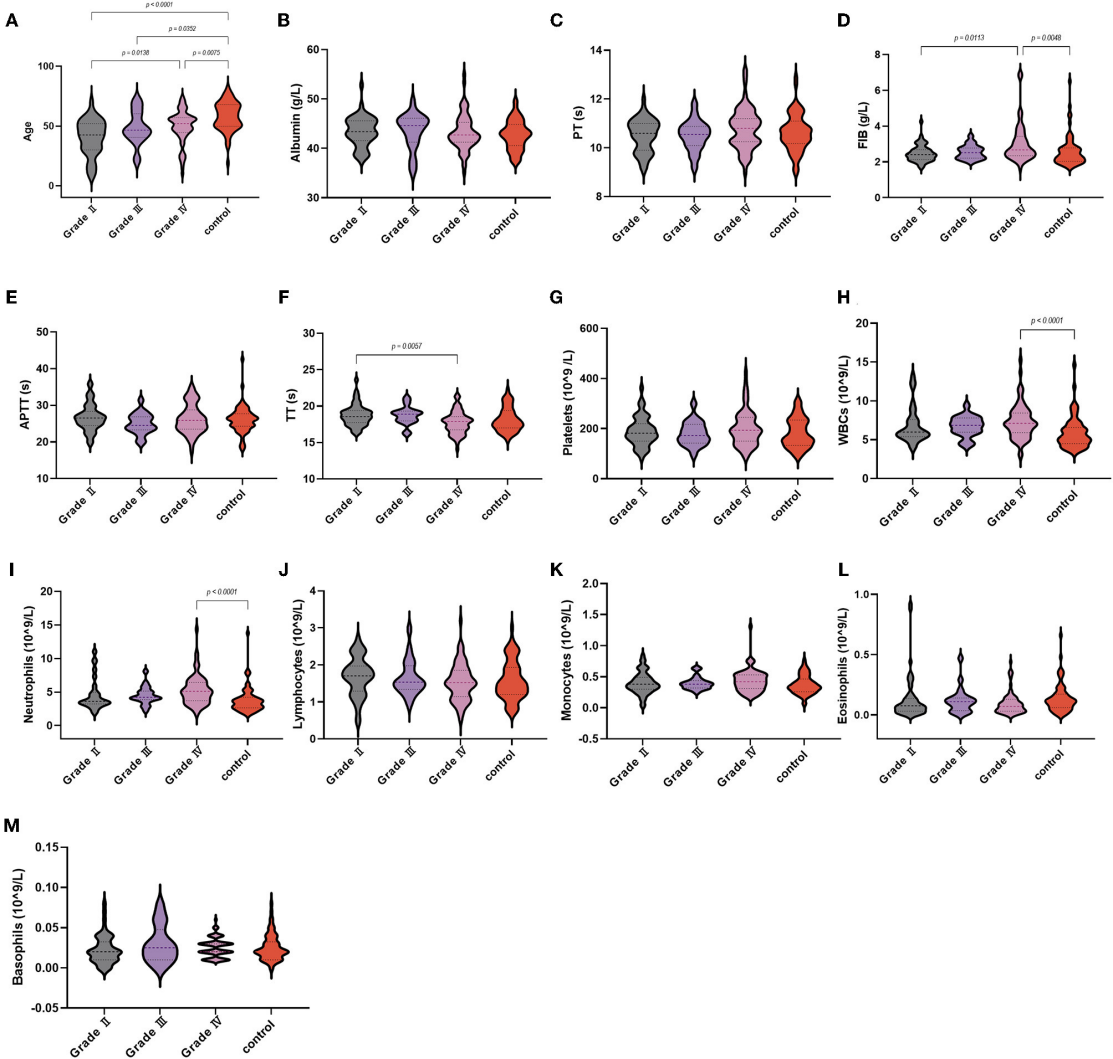
Violin diagram showing comparative results of characteristics in the trigeminal neuralgia group, glioma grade I, glioma grade II, glioma grade III, and glioma grade IV group (the dashed line in the middle represents the median and the dashed lines on both sides represent the interquartile range). **(A)** Age, **(B)** albumin, **(C)** PT, **(D)** FIB, **(E)** APTT, **(F)** TT, **(G)** platelets, **(H)** WBCs, **(I)** neutrophils, **(J)** lymphocytes, **(K)** monocytes, **(L)** eosinophils, **(M)** basophils.

We further analyzed these parameters in different grades of glioma according to the WHO (Table 2). Among the coagulation parameters (Figure 4), the FIB of GBM [2.67 (1.62–6.85)] was significantly higher than the control group and glioma grade II. For inflammation markers, white blood cell counts and neutrophils were both significantly higher in gliomas [7.1 (3–15.26) and 5.1 (1.8–14.37)] than in the control group. Differences in lymphocyte counts, monocytes, eosinophils, and basophil counts were not observed.

**Table 2:** Correlations between preoperative inflammatory markers and glioma grade.

**Marker**	**Trigeminal neuralgia**	**Glioma grade**			
		**I (*****n** =* **1)**	**II (*****n** =* **50)**	**III (*****n** =* **20)**	**IV (*****n** =* **69)**
Age	58.5 (19–82)	44	42.5 (8–71)^*^	46.5 (21–72)^*^	52 (10–74)^*†^
Albumin in g/L	42.9 (37.3–49.6)	41.8	43.4 (37.5–52.8)	44.6 (35.3–49.3)	42.7 (35.1–55)
PT	10.6 (9–12.9)	10.8	1055 (9.2–11.8)	10.55 (9.2–11.8)	10.8 (9–13.2)
FIB	2.35 (1.71–6.5)	2.14	2.41 (1.7–4.25)	2.52 (2–3.44)	2.67 (1.62–6.85)^*†^
APTT	26.2 (18.1–42.6)	28.7	26.55 (19.7–36)	24.5 (19.8–31.4)	25.9 (17.4–34.9)
TT	17.8 (15.3–22.3)	17	18.55 (16.3–23.6)	18.9 (16.2–21.2)	17.9 (14.1–21.4)^†^
Platelets (10^∧^9/L)	166 (88–314)	139	181.5 (83–362)	172 (97–299)	193 (90–426)
WBCs (10^∧^9/L)	5.41 (3.3–14.62)	5.12	5.99 (3.9–13.37)	6.86 (4.29–9.39)	7.1 (3–15.26)^*^
Neutrophils (10^∧^9/L)	3.48 (1.57–13.79)	3.07	3.61 (1.9–11.07)	4.23 (2.24–8.08)	5.1 (1.8–14.37)^*^
Lymphocytes (10^∧^9/L)	1.48 (0.74–3.01)	1.46	1.71 (0.49–2.71)	1.54 (0.95–2.96)	1.52 (0.5–3.19)
Monocytes (10^∧^9/L)	0.34 (0.06–0.77)	0.37	0.38 (0.03–0.84)	0.38 (0.25–0.64)	0.42 (0.17–1.31)
Eosinophils (10^∧^9/L)	0.12 (0–0.66)	0.19	0.08 (0–0.92)	0.11 (0.01–0.47)	0.07 (0–0.44)
Basophils (10^∧^9/L)	0.02 (0–0.08)	0.03	0.02 (0–0.08)	0.03 (0–0.08)	0.02 (0.01–0.06)
NLR	2.11 (0.97–18.39)	2.1	2.09 (0.97–17.31)	2.59 (1.31–8.51)	3.42 (0.98–28.74)^*†^
dNLR	1.32 (1.11–2.32)	1.4	1.3 (1.1–1.75)	1.34 (1.17–1.78)	1.35 (1.15–2.13)
PLR	107.8 (48.81–409.5)	95.21	105.67 (41.25–342.86)	108.9 (61.78–237.89)	128.76 (61.44–776)
LMR	4.42 (0.96–12.5)	3.95	4.35 (1.44–16.33)	4.22 (2.63–7.59)	3.82 (0.92–9)
PNI	50.2 (41–60.6)	49.1	51.78 (44.5–60.3)	52.73 (43.2–61)	50.18 (40.55–66.65)
SII	366.67 (151.31–3934.75)	292.28	397.48 (152.58–2907.43)	476.32 (202.52–1922.19)	646.7 (170.91–3142.07)^*^
PIV	124.8 (26.99–2462)	108.1	154.2 (21.36–1381)	191.5 (50.63–615.1)	243.0 (32.47–4126)^*†^

Values are median (range). ^*^p < 0.05, compared to trigeminal neuralgia. ^∧^p < 0.05, compared to glioma grade I glioma patients. ^†^p < 0.05, compared to glioma grade II glioma patients. ^‡^p < 0.05, compared to glioma grade III glioma patients.

As for laboratory parameters (Figure 3B), NLR and PIV were higher in GBM than in controls and glioma grade II, and SII was higher than in controls. The differences in dNLR, PLR, LMR, and PNI were not significant.

### Correlation of blood markers and their pairs with glioma grade

**Figure 5:**
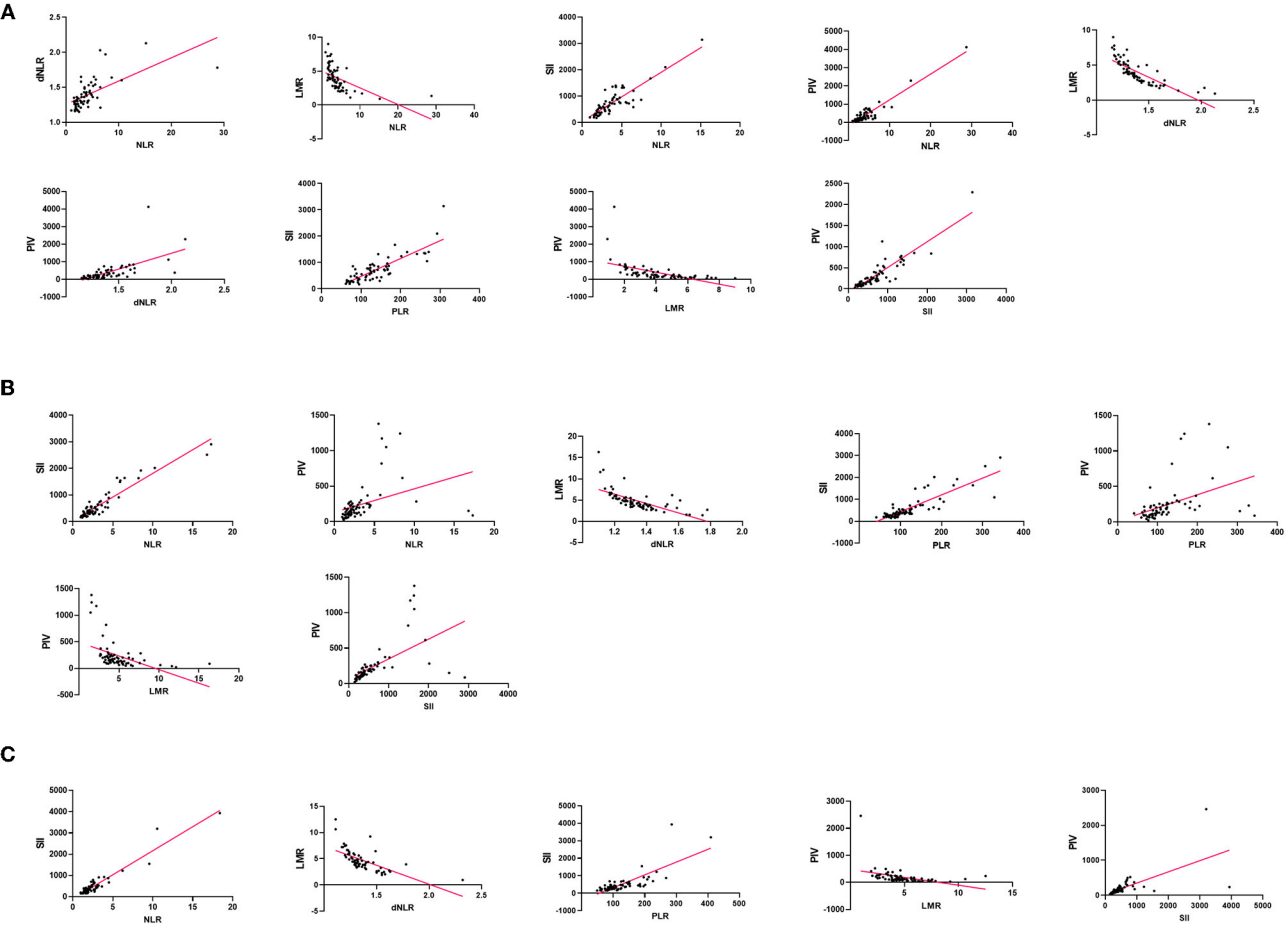
Correlation of blood markers and their pairs: **(A)** in the GBM group, **(B)** in the glioma grade I– III group, **(C)** in the control group.

To study the correlation between laboratory parameters and glioma grade better, we respectively analyzed the correlation among NLR, dNLR, PLR, LMR, PNI, and SII in GBM, glioma grade I–III, and control group (Figure 5, Supplementary Tables S2–S4).

In the GBM group, NLR and SII (*r* = 0.8411, *p* < 0.0001) showed the strongest correlation. In the glioma grade I-III group, PLR and SII (*r* = 0.8376, *p* < 0.0001) showed a significant positive correlation, but little difference with NLR and SII, SII and PIV. SII and PIV (*r* = 0.8778, *p* < 0.0001) is the highest positive correlation in the control group. In contrast, the negative correlation between dNLR and LMR was the strongest in all three groups.

Although NLR and dNLR, NLR and PLR, NLR and SII, NLR and PIV, dNLR and PIV, PLR and SII, PLR and PIV, and SII and PIV were positively correlated in all three groups, the degree of correlation was inconsistent. Among the eight pairs of markers, NLR and dNLR, NLR and SII, NLR and PIV, and dNLR and PIV were higher in the GBM group than the glioma grade I-III group than the control group.

### Diagnostic value of blood markers and their pairs in glioma diagnosis and glioma grading

**Figure 6:**
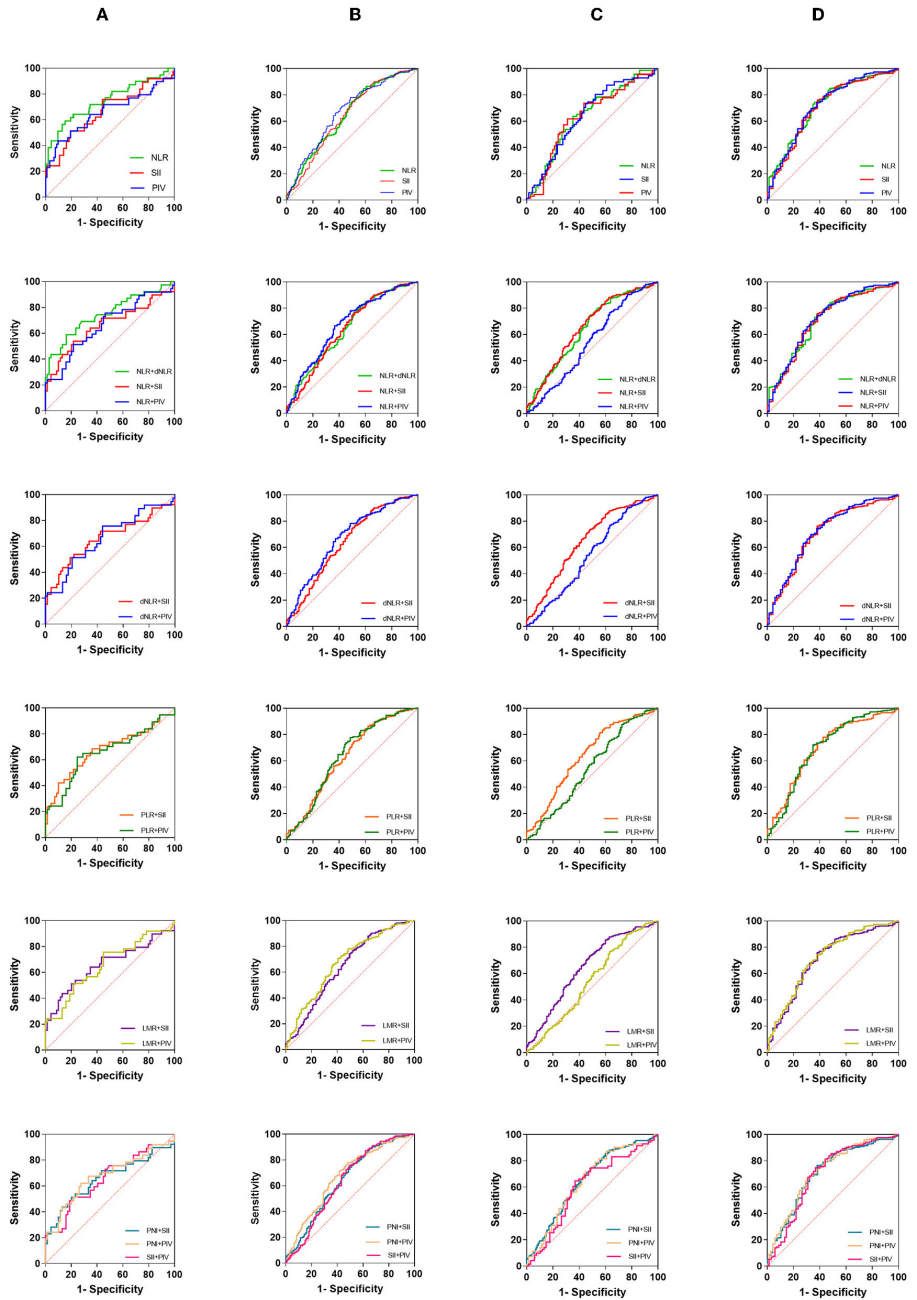
The diagnostic value of preoperative inflammatory markers in glioma diagnosis and glioma grading. **(A)** Acoustic neuroma vs. brain metastases, **(B)** glioma vs. meningioma, **(C)** GBM vs. WHO I–III, **(D)** GBM vs. others (exclude brain metastases).

Since the different performances of these indicators in our various tests, we further evaluated the clinical value of these markers and their pairs (Table 3, Figure 6).

**Table 3:** Diagnostic value of NLR, dNLR, PLR, LMR, PNI, and pairs.

**Marker**	**Acoustic neuroma vs. brain metastases**		**Glioma vs. meningioma**		**GBM vs. WHO I–III**		**GBM vs. others (exclude brain metastases)**	
	**AUC (95% CI)**	***p*** **-value**	**AUC (95% CI)**	***p*** **-value**	**AUC (95% CI)**	***p*** **-value**	**AUC (95% CI)**	***p*** **-value**
NLR	0.7490 (0.6482–0.8498)	< 0.0001^*^	0.6505 (0.5947–0.7068)	< 0.0001^*^	0.6409 (0.5489–0.7330)	0.0040^*^	0.7200 (0.6551–0.7849)	< 0.0001^*^
dNLR	0.6450 (0.5322–0.7577)	0.0090^*^	0.5709 (0.5109–0.6308)	0.0159^*^	0.5706 (0.4758–0.6655)	0.1493	0.5979 (0.5217–0.6740)	0.0078^*^
PLR	0.5972 (0.4843–0.7101)	0.0815	0.5510 (0.4910–0.6109)	0.0828	0.6048 (0.5102–0.6994)	0.0323^*^	0.6071 (0.5330–0.6811)	0.0036^*^
LMR	0.7065 (0.6004–0.8127)	0.0002^*^	0.6300 (0.5716–0.6883)	< 0.0001^*^	0.6137 (0.5200–0.7070)	0.0203^*^	0.6675 (0.5937–0.7412)	< 0.0001^*^
PNI	0.6409 (0.5341–0.7477)	0.0108^*^	0.5061 (0.4487–0.5635)	0.8360	0.5683 (0.4726–0.6640)	0.1633	0.5504 (0.4817–0.6190)	0.1707
SII	0.6589 (0.5434–0.7745)	0.0040^*^	0.6441 (0.5866–0.7015)	< 0.0001^*^	0.6346 (0.5407–0.7285)	0.0062^*^	0.7089 (0.6407–0.7771)	< 0.0001^*^
PIV	0.6646 (0.5533–0.7760)	0.0035^*^	0.6726 (0.6178–0.7274)	< 0.0001^*^	0.6444 (0.5522–0.7367)	0.0032^*^	0.7200 (0.6526–0.7874)	< 0.0001^*^
NLR + dNLR	0.7503 (0.6484–0.8522)	< 0.0001^*^	0.6485 (0.5925–0.7045)	< 0.0001^*^	0.6457 (0.5891–0.7023)	< 0.0001^*^	0.7125 (0.6462–0.7789)	< 0.0001^*^
NLR + PLR	0.5946 (0.4772–0.7120)	0.1036	0.5541 (0.4943–0.6140)	0.0654	0.5705 (0.5114–0.6296)	0.0169^*^	0.6120 (0.5385–0.6855)	0.0023^*^
NLR + LMR	0.6052 (0.4914–0.7189)	0.0703	0.5245 (0.4656–0.5833)	0.4053	0.5476 (0.4899–0.6052)	0.1073	0.5591 (0.4892–0.6291)	0.1079
NLR + PNI	0.5219 (0.4024–0.6414)	0.7059	0.5706 (0.5139–0.6274)	0.0162^*^	0.5615 (0.5046–0.6183)	0.0373^*^	0.5333 (0.4647–0.6019)	0.3651
NLR + SII	0.6607 (0.5447–0.7768)	0.0057^*^	0.6396 (0.5818–0.6973)	< 0.0001^*^	0.6471 (0.5901–0.7040)	< 0.0001^*^	0.6988 (0.6288–0.7689)	< 0.0001^*^
NLR + PIV	0.6627 (0.5497–0.7758)	0.0059^*^	0.6743 (0.6196–0.7289)	< 0.0001^*^	0.5448 (0.4838–0.6059)	0.1291	0.7199 (0.6522–0.7876)	< 0.0001^*^
dNLR + PLR	0.5894 (0.4721–0.7066)	0.1240	0.5510 (0.4910–0.6109)	0.0827	0.5669 (0.5075–0.6262)	0.0236^*^	0.6072 (0.5333–0.6812)	0.0035^*^
dNLR + LMR	0.6659 (0.5550–0.7769)	0.0043^*^	0.6307 (0.5725–0.6890)	< 0.0001^*^	0.6203 (0.5612–0.6794)	< 0.0001^*^	0.6702 (0.5967–0.7437)	< 0.0001^*^
dNLR + PNI	0.6349 (0.5244–0.7454)	0.0202^*^	0.5026 (0.4452–0.5600)	0.9301	0.5050 (0.4472–0.5629)	0.8646	0.5457 (0.4774–0.6139)	0.2140
dNLR + SII	0.6596 (0.5434–0.7758)	0.0060^*^	0.6395 (0.5817–0.6972)	< 0.0001^*^	0.6469 (0.5899–0.7038)	< 0.0001^*^	0.6987 (0.6286–0.7688)	< 0.0001^*^
dNLR + PIV	0.6645 (0.5514–0.7776)	0.0055^*^	0.6737 (0.6190–0.7283)	< 0.0001^*^	0.5443 (0.4833–0.6054)	0.1333	0.7204 (0.6530–0.7878)	< 0.0001^*^
PLR + LMR	0.5851 (0.4674–0.7028)	0.1430	0.5470 (0.4869–0.6071)	0.1095	0.5634 (0.5038–0.6231)	0.0317^*^	0.6029 (0.5280–0.6778)	0.0051^*^
PLR + PNI	0.5723 (0.4529–0.6916)	0.2135	0.5530 (0.4930–0.6130)	0.0712	0.5694 (0.5101–0.6287)	0.0188^*^	0.6060 (0.5314–0.6805)	0.0039^*^
PLR + SII	0.6570 (0.5401–0.7740)	0.0069^*^	0.6347 (0.5765–0.6929)	< 0.0001^*^	0.6458 (0.5888–0.7028)	< 0.0001^*^	0.7032 (0.6351–0.7712)	< 0.0001^*^
PLR + PIV	0.6573 (0.5406–0.7739)	0.0078^*^	0.6440 (0.5853–0.7028)	< 0.0001^*^	0.5505 (0.4897–0.6113)	0.0871	0.7002 (0.6271–0.7732)	< 0.0001^*^
LMR + PNI	0.6590 (0.5499–0.7682)	0.0062^*^	0.5382 (0.4804–0.5960)	0.1937	0.5417 (0.4834–0.5999)	0.1583	0.5974 (0.5269–0.6678)	0.0081^*^
LMR + SII	0.6589 (0.5427–0.7751)	0.0063^*^	0.6390 (0.5812–0.6968)	< 0.0001^*^	0.6467 (0.5898–0.7037)	< 0.0001^*^	0.6985 (0.6283–0.7686)	< 0.0001^*^
LMR + PIV	0.6580 (0.5443–0.7718)	0.0075^*^	0.6745 (0.6201–0.7290)	< 0.0001^*^	0.5437 (0.4826–0.6047)	0.1391	0.7206 (0.6536–0.7875)	< 0.0001^*^
PNI + SII	0.6574 (0.5406–0.7741)	0.0068^*^	0.6390 (0.5812–0.6968)	< 0.0001^*^	0.6474 (0.5904–0.7043)	< 0.0001^*^	0.6989 (0.6288–0.7691)	< 0.0001^*^
PIN + PIV	0.6547 (0.5406–0.7688)	0.0088^*^	0.6739 (0.6192–0.7287)	< 0.0001^*^	0.6521 (0.5955–0.7087)	< 0.0001^*^	0.7187 (0.6512–0.7862)	< 0.0001^*^
SII + PIV	0.6757 (0.5605–0.7909)	0.0030^*^	0.6374 (0.5779–0.6969)	< 0.0001^*^	0.6060 (0.5106–0.7015)	0.0310^*^	0.6991 (0.6243–0.7738)	< 0.0001^*^

Others include patients with trigeminal neuralgia, craniopharyngioma, ependymoma, acoustic neuroma, meningioma, and glioma grade I–III. ^*^P < 0.05.

When the acoustic neuroma group was compared with the brain metastatic tumor group (Figure 6A), NLR [0.7490 (0.6482–0.8498)] and NLR + dNLR [0.7481 (0.6457–0.8505)] performed well, but there was little difference between NLR single and paired. When glioma was compared with meningioma (Figure 6B), our results showed that NLR [0.6505 (0.5947–0.7068)] ranked second, next to PIV [0.6726 (0.6178–0.7274)]. NLR + PIV [0.6743 (0.6196–0.7289)], LMR + PIV [0.6745 (0.6201–0.7290)], and PIN + PIV [0.6739 (0.6192–0.7287)] showed higher AUCs. When GBM was compared with glioma grade I– III (Figure 6C), most markers showed lower AUCs overall. Among them, PIV [0.6444 (0.5522–0.7367)] and NLR + SII [0.6565 (0.5999–0.7130)] are relatively higher. When GBM was compared with other brain tumors (excluding brain metastases), NLR [0.7200(0.6551–0.7849)] and PIV [0.7200 (0.6526–0.7874)] performed similarly. Here, a number of markers and pairs show higher diagnostic predictive value, such as NLR + PIV [0.7199 (0.6522–0.7876], dNLR + PIV [0.7204 (0.6530–0.7878)], and PNI + PIV [0.7187 (0.6512–0.7862)]. Among the combined parameters, only NLR + LMR, NLR + PNI, and dNLR + PNI were not significant.

Regrettably, PNI did not perform well enough. In general, NLR, SII, PIV, and their pairing gave remarkable results, which showed significant predictive value in all subgroups.

## Discussion

A growing number of studies have shown that inflammation is clearly present in the early stages of tumor progression, which may promote tumor progression and lead to poor prognosis (7, 20, 21). Peripheral blood biomarkers such as NLR and PLR have attracted widespread attention, but the discriminatory ability of such single biomarkers has always been limited. For this reason, excluding factors that may affect coagulation parameters, inflammatory markers, and albumin in the blood, we analyzed all previously reported parameters (NLR, dNLR, PLR, LMR, PNI, SII, and PIV) as comprehensively as possible and aimed to find the most specific and accurate blood-based biomarkers.

Whether the thrombotic disease without foundation is a clinical marker of occult cancer has been controversial (22, 23). Recently, it has also been reported that venous thromboembolism usually occurs shortly after diagnostic surgery for glioma (24). Therefore, in the present study, we are also concerned with partial coagulation parameters and speculate whether tumors could be detected early by abnormal coagulation parameters. Our data show that FIB levels were elevated in the GBM group relative to patients with trigeminal neuralgia and glioma grade II patients. At the same time, TT was shorter in the GBM group. FIB is a sensitive biochemical index, and its increase reflects not only an imbalance of coagulation or fibrinolytic system but also systemic inflammatory syndrome when inflammation is present in the body. Because of this, abnormal coagulation parameters, including FIB, may contribute to the determination of the malignancy of glioma but may also be related to the inflammatory response of the organism caused by cancer. Thus, we need more studies to discuss this issue.

In this study, significantly elevated white blood cell counts were observed in glioma patients compared to patients with trigeminal neuralgia, acoustic neuroma, and meningioma. Although white blood cell count is not currently considered a blood marker for glioma, elevated neutrophil counts have long been associated with tumor growth. Many studies about neutrophils have focused on angiogenesis, a characteristic of high-grade gliomas (25, 26). Our data also suggest that glioma had a higher neutrophil count than trigeminal neuralgia, acoustic neuroma, and meningioma. Some researchers have proposed that neutrophils, on the one hand, inhibit the anticancer activity of other immune cells by releasing reactive oxygen species (ROS) (27, 28), thus promoting tumor occurrence; on the other hand, they promote tumor proliferation and combat tumor cell senescence through various paracrine signaling pathways (29).

NLR has been confirmed to be associated with glioma prognosis in several studies (8–10). The almost overwhelming data also suggest that NLR is associated with glioma identification and grading (14, 15). NLR also ranked first in our study with an AUC of 0.7490 (0.6482–0.8498). The combination of NLR and dNLR ranked second, which strongly confirmed their predictive ability. SII levels provide prognostic evidence for many solid tumors, such as prostate cancer (30), breast cancer (31), and gastric cancer (32). In our results, SII levels were significantly elevated in brain metastases and GBM. When compared with brain tumors other than brain metastases, SII and all combinations with it are highly accuracy in GBM, including NLR + SII, dNLR + SII, PLR + SII, LMR + SII, and PNI + SII. Consistent with the results of a recent meta-analysis (33), the strong discriminatory potential of SII for malignancies was also demonstrated in this study. The accuracy of PIV and all its pairs is higher. The strong correlation between PIV and the other five indicators also indicates that PIV has more diagnostic value when combined. As in most studies, differences in platelet count and lymphocyte count were not significant in the classification of brain tumors and the grading of gliomas (15, 34). In parallel, the changes in dNLR were not significant in our study. Therefore, we prefer that the changes in the levels of these laboratory parameters be mainly reflected in the elevation of neutrophils. Significant differences were mainly concentrated in brain metastases and gliomas compared with other non-malignant tumors, so we believe that the results of this study will be more helpful in judging the malignancy of brain tumors. The significant difference between glioma and other tumors stems from the highly malignant characteristics of GBM.

## Limitations

In addition, there are some limitations in our study: (1) Small sample types and numbers. Among the patients included in our study, craniopharyngioma, ependymoma, chordoma, and glioma grade I samples were small, and other brain tumors, such as pituitary tumors and lymphomas, were not included. (2) The case group is quite heterogeneous, which may lead to bias. (3) Lack of healthy human samples. We had to use trigeminal neuralgia samples as a control group for brain tumors, but we cannot exclude that trigeminal neuralgia disease itself causes alterations in these markers. (4) A single combination approach. We only use addition to combine the various indicators and more combinations that deserve subsequent exploration. (5) False-positive may exist. A lot of positive results in our study, but there are many factors that we are not aware of that could be contributing to this result.

## Conclusion

In summary, our data suggest that NLR, SII, PIV, and their pairs are promising biomarkers to help determine tumor type, grade, and malignancy degree of brain tumors. Additionally, larger samples and more categorized studies should be conducted for clinical practice.

## Data availability statement

The raw data supporting the conclusions of this article will be made available by the authors, without undue reservation.

## Ethics statement

The requirement of ethical approval was waived by the General Hospital of Western Theater Command for the studies involving humans because of the retrospective nature of the study and due to all procedures being performed as part of routine care. The studies were conducted in accordance with the local legislation and institutional requirements. Written informed consent for participation in this study was provided by the participants' legal guardians/next of kin.

## Author contributions

YY: Data curation, Writing—original draft, Writing—review and editing. FH: Data curation, Writing—original draft. SW: Data curation, Writing—original draft. ZH: Data curation, Writing—original draft. KW: Data curation, Writing—original draft. YM: Funding acquisition, Methodology, Writing—review and editing. QO-Y: Funding acquisition, Methodology, Writing—review and editing.
